# Large Hydrogen Isotope Fractionation Distinguishes Nitrogenase-Derived Methane from Other Methane Sources

**DOI:** 10.1128/AEM.00849-20

**Published:** 2020-09-17

**Authors:** Katja E. Luxem, William D. Leavitt, Xinning Zhang

**Affiliations:** a Department of Geosciences, Princeton University, Princeton, New Jersey, USA; b Princeton Environmental Institute, Princeton University, Princeton, New Jersey, USA; c Department of Earth Sciences, Dartmouth College, Hanover, New Hampshire, USA; d Department of Chemistry, Dartmouth College, Hanover, New Hampshire, USA; e Department of Biological Sciences, Dartmouth College, Hanover, New Hampshire, USA; Kyoto University

**Keywords:** *Rhodopseudomonas palustris*, methane, nitrogenase, stable isotopes

## Abstract

Biological nitrogen fixation is catalyzed by the enzyme nitrogenase. Two forms of this metalloenzyme, the vanadium (V)- and iron (Fe)-only nitrogenases, were recently found to reduce small amounts of carbon dioxide (CO_2_) into the potent greenhouse gas methane (CH_4_). Here, we report carbon (^13^C/^12^C) and hydrogen (^2^H/^1^H) stable isotopic compositions and fractionations of methane generated by V- and Fe-only nitrogenases in the metabolically versatile nitrogen fixer Rhodopseudomonas palustris. The stable carbon isotope fractionation imparted by both forms of alternative nitrogenase are within the range observed for hydrogenotrophic methanogenesis (^13^α_CO2/CH4_ = 1.051 ± 0.002 for V-nitrogenase and 1.055 ± 0.001 for Fe-only nitrogenase; values are means ± standard errors). In contrast, the hydrogen isotope fractionations (^2^α_H2O/CH4_ = 2.071 ± 0.014 for V-nitrogenase and 2.078 ± 0.018 for Fe-only nitrogenase) are the largest of any known biogenic or geogenic pathway. The large ^2^α_H2O/CH4_ shows that the reaction pathway nitrogenases use to form methane strongly discriminates against ^2^H, and that ^2^α_H2O/CH4_ distinguishes nitrogenase-derived methane from all other known biotic and abiotic sources. These findings on nitrogenase-derived methane will help constrain carbon and nitrogen flows in microbial communities and the role of the alternative nitrogenases in global biogeochemical cycles.

**IMPORTANCE** All forms of life require nitrogen for growth. Many different kinds of microbes living in diverse environments make inert nitrogen gas from the atmosphere bioavailable using a special enzyme, nitrogenase. Nitrogenase has a wide substrate range, and, in addition to producing bioavailable nitrogen, some forms of nitrogenase also produce small amounts of the greenhouse gas methane. This is different from other microbes that produce methane to generate energy. Until now, there was no good way to determine when microbes with nitrogenases are making methane in nature. Here, we present an isotopic fingerprint that allows scientists to distinguish methane from microbes making it for energy versus those making it as a by-product of nitrogen acquisition. With this new fingerprint, it will be possible to improve our understanding of the relationship between methane production and nitrogen acquisition in nature.

## Abstract (precis)

All forms of life require nitrogen for growth. Many different kinds of microbes living in diverse environments make inert nitrogen gas from the atmosphere bioavailable using a special enzyme, nitrogenase. Nitrogenase has a wide substrate range, and, in addition to producing bioavailable nitrogen, some forms of nitrogenase also produce small amounts of the greenhouse gas methane. This is different from other microbes that produce methane to generate energy. Until now, there was no good way to determine when microbes with nitrogenases are making methane in nature. Here, we present an isotopic fingerprint that allows scientists to distinguish methane from microbes making it for energy versus those making it as a by-product of nitrogen acquisition. With this new fingerprint, it will be possible to improve our understanding of the relationship between methane production and nitrogen acquisition in nature.

## INTRODUCTION

Microorganisms produce over half of global methane (CH_4_) emissions (1). Fermentative and hydrogenotrophic methanogens are the most significant microbial producers of this potent greenhouse gas (1, 2). Their metabolic pathways occur exclusively within anaerobic *Archaea* and involve multiple enzymes working together in series, including the obligatory methyl-coenzyme M reductase (Mcr). The primary function of Mcr is for catabolism, with methane production thought to occur only after other more favorable electron acceptors, like oxygen, nitrate, or sulfate, have been consumed (3–5). Over the past decade, it has been recognized that minor additional contributions of methane derive from the demethylation of organophosphonates (6–8) and from inefficient Wood-Ljungdahl pathway carbon fixation (9). Most recently, it was discovered that some forms of the metalloenzyme nitrogenase also reduce carbon dioxide (CO_2_) into methane (10). The discovery of biological methane production by certain forms of nitrogenase expands the known range of organisms and environments in which methane production is possible.

Nitrogenase is known primarily for its ability to reduce inert dinitrogen (N_2_) gas to ammonia, a process known as nitrogen fixation. It is the only enzyme that can catalyze the production of newly fixed nitrogen for the biosphere. Prior to industrial reduction of dinitrogen, biological nitrogen fixation catalyzed by nitrogenase was the primary source of nitrogen for life on Earth (11, 12). Nitrogenase is generally considered a promiscuous enzyme because it can reduce a variety of carbon-containing compounds in addition to N_2_ (13–17). For example, the iron (Fe)-only nitrogenase isoform can convert carbon monoxide into hydrocarbon chains, a reaction that may have been important for early forms of life (15). In addition, all forms of nitrogenase reduce acetylene to ethylene (18–21). This is the basis for the acetylene reduction assay, the most commonly used method to measure nitrogen fixation rates in the laboratory and field (22–25). The recent discovery that some forms of nitrogenase can reduce carbon dioxide to methane (10) is significant because, unlike acetylene and carbon monoxide, carbon dioxide is ubiquitous in nature.

The vanadium (V)- and Fe-only nitrogenases produce the most by-product methane of the nitrogenase isoforms (10). They are found in both the bacterial and archaeal domains and are present in diverse environments (26–31). In addition, certain artificial mutations near the active site of the molybdenum (Mo)-nitrogenase enable this more common isoform to produce methane as well (32, 33). These findings beg the question of whether and to what extent carbon dioxide reduction by nitrogenase is an important methane source in certain environments and how to distinguish nitrogenase-derived methane from other sources. The stable isotopes of carbon (^13^C/^12^C) and hydrogen (^2^H/^1^H) are commonly used to differentiate (fingerprint) different sources of methane (2, 8, 35–40). Previous research has established that each form of nitrogenase imparts a characteristic nitrogen or carbon isotope fractionation during N_2_ (34) or acetylene (27) reduction, respectively. To determine what characteristic carbon and hydrogen isotope fractionations are associated with methane production by the different nitrogenases, we cultivated V- and Fe-only nitrogenase-utilizing strains of the anoxygenic photoheterotroph Rhodopseudomonas palustris under nitrogen-fixing conditions. We find that the carbon isotope fractionations are large yet similar to those of canonical anaerobic methanogens. Conversely, the hydrogen isotope fractionation values are the largest of any methane production pathway on record. This unique hydrogen isotopic fingerprint allows us to differentiate nitrogenase-derived methane from methane generated by other physiological pathways and provides a new tool to gain insight into the mechanism of proton delivery to nitrogenase.

## RESULTS AND DISCUSSION

### Isotope fractionation by nitrogenase during methane production.

**FIG 1:**
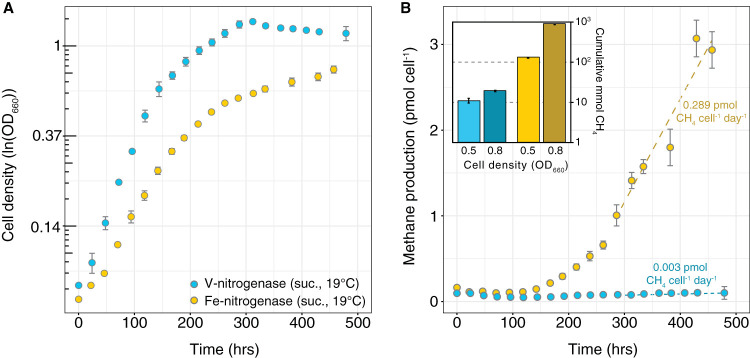
Growth dynamics (A) and methane yields (B) of nitrogenase strains. During growth on succinate (suc.) at 19°C (A), the R. palustris V- and Fe-only nitrogenase strains produced methane (B). The Fe-only nitrogenase strain produced >10-fold more methane in the headspace than the V-nitrogenase strain. For the Fe-only nitrogenase strain, methane production per cell is greater at higher cell densities. Error bars show the standard errors from three biological replicates. Dissolved methane is not included.

Different methane sources are commonly associated with characteristic stable isotope fractionations that can help distinguish between different biogenic, geogenic, and thermogenic sources (2, 37, 40). To determine the stable isotopes associated with methane production by nitrogenase, we grew mutant strains of the anoxygenic photoheterotroph Rhodopseudomonas palustris CGA009 that exclusively utilize either the Mo-nitrogenase, V-nitrogenase, or Fe-only nitrogenase for nitrogen fixation (10, 41, 42). The Mo-nitrogenase strain did not produce detectable methane during batch culture incubation through stationary phase in Balch tubes (data not shown). The V- and Fe-only nitrogenase strains both produced methane, with the Fe-only nitrogenase strain producing over an order of magnitude more methane than the V-nitrogenase strain (Fig. 1). For the Fe-only nitrogenase strain, methane production per cell was higher later during growth. We measured the carbon (^13^C/^12^C) and hydrogen (^2^H/^1^H) isotopic compositions of methane and fractionations relative to carbon dioxide (CO_2_/CH_4_) and water (H_2_O/CH_4_), as produced by the V- and Fe-only nitrogenases across a range of cell densities (optical density at 660 nm of ∼0.3 to 1.3, from early log to stationary phase), temperatures (14 to 30°C), carbon substrates (succinate and acetate), and growth medium pH (from 6.2 to 6.8 at inoculation).

**FIG 2:**
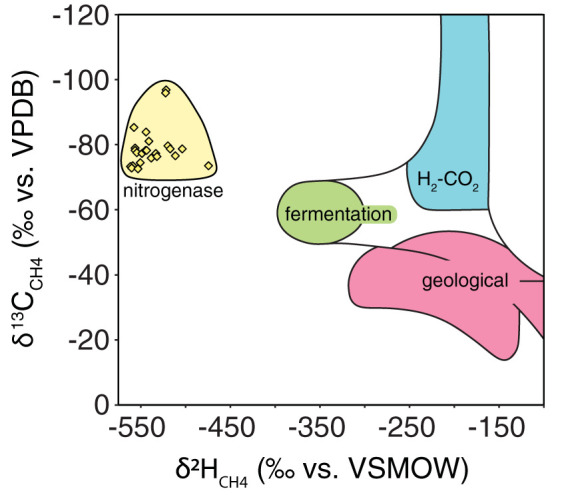
Nitrogenase-derived methane has a unique isotopic composition. The stable isotopic composition of methane produced by nitrogenase (yellow) can be distinguished from other natural methane sources due to its more depleted hydrogen isotopic composition. Individual data points from this study are shown as diamonds (*n* = 31). The observed ranges for fermentative (green) and hydrogenotrophic (blue) *mcr*-based methanogenesis pathways and geological methane sources (red) were taken from reference 93, although we note that these boundaries are not absolute (see, e.g., reference 37).

We discovered that methane produced by the V- and Fe-only nitrogenases is highly depleted in deuterium (^2^H) relative to other natural sources (Fig. 2). Growth in water with δ^2^H = 1,000 × [(^2^H/^1^H_water_)/(^2^H/^1^H_VSMOW_) − 1] of ∼−40‰ yielded methane with δ^2^H values ranging from −473 to −560‰. To our knowledge, this is the most deuterium-depleted hydrogen isotope ratio measured for biogenic methane sources to date. The methane carbon isotopic composition, which varied from δ^13^C = 1,000 × [(^13^C/^12^C_CH4_)/(^13^C/^12^C_VPDB_) − 1] = −73.0 to −97.1‰ for substrate CO_2_ of ∼−30‰, falls within the range observed for hydrogenotrophic methanogenesis (2) but is distinct from methane generated by other abiogenic (37) and nontraditional biotic pathways (8).

**FIG 3:**
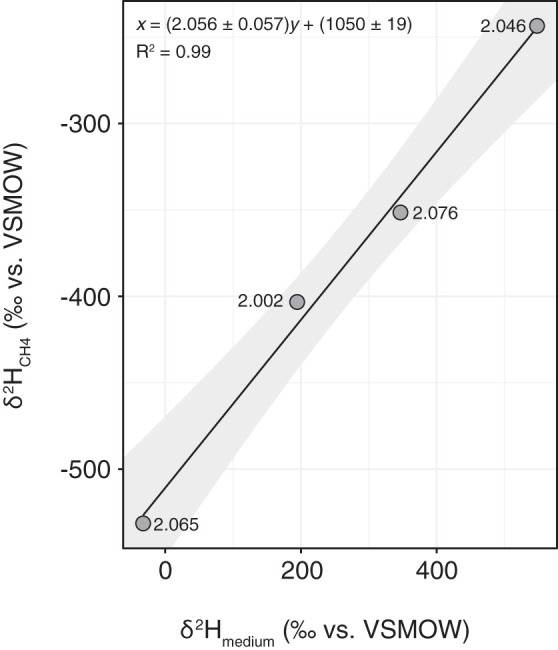
Hydrogen isotope fractionation does not depend on water isotopic composition. Regression of δ^2^H values for source water versus methane shows that hydrogen isotope fractionation (^2^α_H2O/CH4_) is constant over a 600‰ range for the Fe-only nitrogenase strain grown at 19°C on succinate. The hydrogen isotope fractionation calculated using the slope (^2^α_H2O/CH4_ = 2.056 ± 0.057, means ± SE), intercept (^2^α_H2O/CH4_ = 1,050/1,000 + 1 ± 19/1,000 = 2.050 ± 0.019), and individual samples (^2^α_H2O/CH4_ = 2.047 ± 0.016) is indistinguishable (*P ≥ *0.9). The values next to each data point are the calculated fractionations for individual samples, and the shaded area shows the 95% confidence interval for the regression. The convention used for individual samples is for substrate over product, whereas the regression line was calculated as product over substrate. The regression calculated for substrate over product, *x* = (2.043 ± 0.117)*y* + (1045 ± 46), is statistically indistinguishable (*P ≥ *0.9).

**FIG 4:**
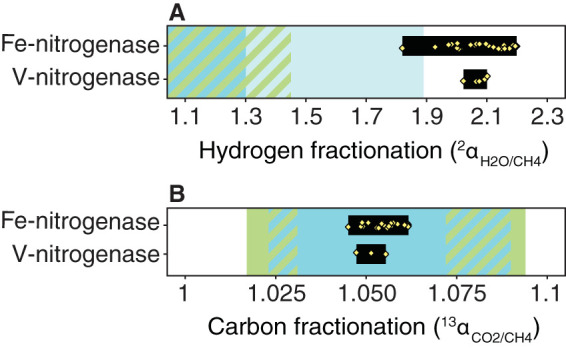
Nitrogenase-derived methane has a characteristic hydrogen isotope fractionation. (A) The largest hydrogen isotope fractionations observed for canonical, *mcr*-based anaerobic methanogenesis pathways, around ∼1.45 (35, 43), are substantially smaller than the hydrogen isotope fractionation observed for nitrogenase, although we note that values as high as 1.89 were observed in one pure-culture study with hydrogenotrophic methanogens (44, 48). (B) In contrast, carbon isotope fractionation by nitrogenase falls within the range observed for hydrogenotrophic methanogenesis (blue, 1.023 ≤ ^13^α_CO2/CH4_ ≤ 1.090) and is intermediate to the range observed for methanol (1.072 ≤ ^13^α_CO2/CH4_ ≤ 1.094)- and acetate (1.017 ≤ ^13^α_CO2/CH4_ ≤ 1.031)-based fermentative methanogenesis (green, 8). Green-blue hatched areas represent ranges of overlap between the fractionations observed for fermentative and hydrogenotrophic methanogenesis. The black bars represent the range of fractionation values measured in this study, with individual data points shown as yellow diamonds.

**FIG 5:**
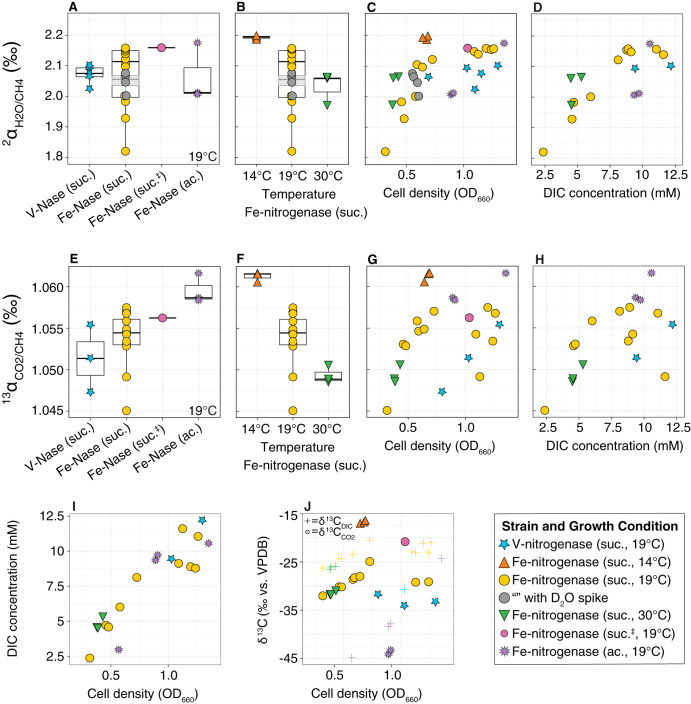
Carbon and hydrogen isotope fractionations associated with methane production by nitrogenase under different growth conditions. Hydrogen and carbon isotope fractionations increase at low temperatures (B and F), higher cell densities (C and G), and higher substrate concentrations (D and H). Hydrogen isotope fractionation is comparable during growth on different organic carbon substrates (succinate, suc.; acetate, ac.) and when the growth medium is acidified (suc.^‡^; A). Carbon isotope fractionation is slightly higher during growth on acetate than on succinate (E), although this could also be related to differences in the cell density at harvest (G). The dissolved inorganic carbon (DIC) concentration (I) and inorganic carbon isotopic composition (J) increase throughout exponential growth, suggesting that substrate concentration influences the observed effect of cell density (OD_660_) on fractionation. The boxplots in panels A, B, E, and F show the median (center line), first and third quartiles (outer lines), and values within 1.5 times the inner quartile range (whiskers). For panel J, note that, for some samples, only δ^13^C_CO2_ or δ^13^C_DIC_ was measured, such that ^13^C_CO2_ or δ^13^C_DIC_ data points are not necessarily paired.

Methane isotope ratios can more reliably be attributed to specific pathways when the isotopic composition of source water and carbon are also considered (35, 37). In our experiments, manipulation of growth medium δ^2^H over a 600‰ range, from −30 to 550‰, resulted in a constant, statistically indistinguishable fractionation of ^2^α_H2O/CH4_ = (δ^2^H_H2O_ + 1,000)/(δ^2^H_CH4_ + 1,000) = 2.047 ± 0.016 calculated for individual samples, ^2^α_H2O/CH4_ = 2.056 ± 0.057 calculated using the slope, and ^2^α_H2O/CH4_ = 2.050 ± 0.019 calculated using the intercept (*P = *0.9) (Fig. 3). The hydrogen isotope fractionations (1.820 ≤ ^2^α_H2O/CH4_ ≤ 2.199) measured for methane production by V- and Fe-only nitrogenase over a range of temperatures and growth conditions are substantially higher than the largest fractionations observed for traditional microbial methanogenesis pathways, which are around ^2^α_H2O/CH4_ of ∼1.45 for acetoclastic (43) and hydrogenotrophic (35) methanogenesis (Fig. 4A and 5). Values as large as ^2^α_H2O/CH4_ = 1.89 have been observed in one pure-culture experiment with a hydrogenotrophic methanogen (44), but in general, depending on substrate concentrations and environmental conditions, the hydrogen isotope fractionation for these traditional methane-forming pathways is often even lower than ^2^α_H2O/CH4_ = 1.45 (35, 43, 45). Our data indicate that a large hydrogen isotope fractionation of ^2^α_H2O/CH4_ = 2.1 is characteristic of methane production by nitrogenase and distinguishes methane produced by nitrogenase from other biogenic and abiogenic pathways.

Like the carbon isotopic composition, the carbon isotope fractionation measured for nitrogenase, 1.045 ≤ ^13^α_CO2/CH4_ = (δ^13^C_CO2_ + 1,000)/(δ^13^C_CH4_ + 1,000) ≤ 1.062, falls within the range observed for hydrogenotrophic methanogenesis (1.030 ≤ ^13^α_CO2/CH4_ ≤ 1.080) (38) (Fig. 4B). It is possible that the similarity in carbon isotope fractionation between these two pathways is due to the similarity in substrate (CO_2_) and the eight-electron requirement of carbon dioxide reduction to methane.

We observed only small changes in nitrogenase fractionation across a large range of temperatures, cell densities, and carbon substrates (<0.38 for ^2^α_H2O/CH4_ and 0.02 for ^13^α_CO2/CH4_) relative to the variability observed for other methane production pathways. Fractionation increased by 0.01 as temperature decreased from 30 to 14°C for ^13^α_CO2/CH4_ (*P = *10^−5^) and by 0.16 for ^2^α_H2O/CH4_ (*P = *0.03) (Fig. 4 and 5). In contrast, the form of growth substrate (succinate or acetate) did not alter ^2^α_H2O/CH4_ (*P = *0.96) and only had a small impact of ∼0.005 on ^13^α_CO2/CH4_ (*P = *0.006). This is compatible with the recent observation that electron availability has only a minor impact on methane production by a mutant Mo-nitrogenase isoform (46). Acidification of the growth medium by ∼0.5 pH unit also did not alter fractionation, although we note that there was only one biological replicate for the acidified treatment (Table 1). Despite more than an order of magnitude difference in the rate of methane production by V- and Fe-only nitrogenase (Fig. 1), they have indistinguishable fractionation factors associated with methane production (*P* = 0.9 for ^2^α_H2O/CH4_ and 0.4 for ^13^α_CO2/CH4_) (Table 1). This suggests that there is no rate effect on fractionation and that the V- and Fe-only nitrogenases share a common mechanism for carbon dioxide reduction to methane.

**TABLE 1:** Carbon and hydrogen stable isotope fractionation associated with methane production by V- and Fe-only nitrogenase^*a*^

Temp	C substrate	^13^α_CO2/CH4_	^13^ε_CO2/CH4_	^13^n	^2^α_H2O/CH4_	^2^ε_H2O/CH4_	^2^n
Fe-only nitrogenase							
14°C	Suc.	1.061 ± 0.001	61.3 ± 0.3‰	3	2.193 ± 0.004	1,193 ± 4‰	3
19°C	Suc.	1.054 ± 0.001	53.7 ± 1.0‰	12	2.063 ± 0.024	1,063 ± 24‰	16
	Suc.^‡^	1.056	56.3‰	1	2.159	1,159‰	1
	Ac.	1.060 ± 0.001	59.6 ± 1.0‰	3	2.064 ± 0.055	1,064 ± 55‰	3
30°C	Suc.	1.049 ± 0.001	49.3 ± 0.6‰	3	2.033 ± 0.030	1,033 ± 30‰	3
All conditions		1.055 ± 0.001	55.1 ± 1.0‰	22	2.078 ± 0.018	1,078 ± 18‰	26
V-nitrogenase							
19°C	Suc.	1.051 ± 0.002	51.4 ± 2.3‰	3	2.071 ± 0.014	1,071 ± 14‰	5

a Mean values ± standard errors. Individual data points, including product and substrate isotopic compositions, are shown in Supplemental File 2. Suc., succinate; Ac., acetate; Suc.‡, acidified.

The greatest source of variability in fractionation (∼0.25 for ^2^α_H2O/CH4_ and ∼0.01 for ^13^α_CO2/CH4_) is due to cell density, growth phase (Fig. 5C and G), or substrate (CO_2_) concentration (Fig. 5D and H). These variables are strongly correlated due to dissolved inorganic carbon (DIC) production throughout growth (Fig. 5I) and cannot be disentangled with the current data set. The DIC concentrations in our experiments, from 2.4 to 12.2 mM at harvest, fall at the higher end of concentrations observed in natural environments (typically 0.1 to 5 mM in rivers and lakes, averaging around 1 mM [47]). Linear extrapolation of the trends in Fig. 5D and H suggest that if the effect is due to DIC concentration rather than growth phase, at lower DIC concentrations, hydrogen and carbon isotope fractionation could be as low as ∼1.7 for ^2^α_H2O/CH4_ and ∼1.038 for ^13^α_CO2/CH4_ (see the discussion in Supplemental File 1). Future experiments are necessary to test whether the observed trend is caused by DIC concentration or growth phase and how this influences the variability of the nitrogenase isotopic fingerprint in natural ecosystems. Notably, a similar cell density or growth phase effect was previously observed for *mcr*-based methanogenesis, where it has been tentatively attributed to changes in temperature, catabolic rate (43, 44, 48), or carbon assimilation during logarithmic growth (49).

The methane isotopic composition at harvest integrates the isotopic composition of methane produced throughout growth. Therefore, the fractionation measured at stationary phase is altered by the change observed in substrate CO_2_ isotopic composition during exponential phase (Fig. 5J). Using the observed shift in medium CO_2_ isotopic composition to estimate the effect on the fractionation measured at stationary phase, we find that the change in substrate isotopic composition could account for at most half (∼0.005) of the total (∼0.01) shift observed in ^13^α_CO2/CH4_ with cell density (see the supplemental material). We note that it is possible that the isotopic composition of intracellular CO_2_ is somewhat different from the bulk composition due to localized production, consumption, and depletion, given the competing reactions of CO_2_ production during organic substrate assimilation and RuBisCO refixation during photoheterotrophic growth of R. palustris (42, 50, 51).

Prior studies of isoform-specific fractionation have shown that ^15^N/^14^N fractionation during N_2_ reduction into biomass and ^13^C/^12^C fractionation during acetylene into ethylene are remarkably constant across different organisms, metabolisms, and environmental conditions, varying by little more than a per mille under the conditions tested so far (27, 34, 95; Darnajoux et al., unpublished data, and Luxem et al., unpublished data). Here, we observed changes of ∼20‰ (carbon) and ∼380‰ (hydrogen) in fractionation by nitrogenase correlated with temperature, growth phase, and DIC concentration but not with organic carbon substrate, total methane production rate, or nitrogenase isoform. This variability could help elucidate the mechanism responsible for the large hydrogen isotope fractionation during methane production by nitrogenase. Even when taking this variability into account, the range of measured hydrogen isotope fractionation is easily distinguished from the range observed for other methane production pathways (Fig. 4).

### Hydrogen concentration does not influence methane isotope fractionation by nitrogenase.

Molecular hydrogen (H_2_) is an obligatory product of nitrogen fixation and, in our experiments, is generated simultaneously with the production of methane from carbon dioxide (52, 53). We explored whether the buildup and isotopic composition of H_2_ influence methane isotope fractionation by nitrogenase, as has been observed for *mcr*-based methanogenesis (2, 43, 48, 54–63).

Two lines of evidence show that the presence of H_2_ does not alter the isotopic composition of methane produced by nitrogenase. First, for Fe-only nitrogenase cultures (grown on succinate at 19°C in serum vials), the hydrogen isotope fractionations were indistinguishable in cultures in which the headspace contained 2 to 3% H_2_ at inoculation (^2^α_H2O/CH4_ = 2.068 ± 0.033, *n* = 3) and in cultures that were flushed with 100% N_2_ prior to inoculation (^2^α_H2O/CH4_ = 2.046 ± 0.016, *n* = 4, *P = *0.57; Supplemental File 2). Although we were not able to measure the δ^2^H of the exogenous H_2_, these data suggest that its presence did not influence the isotopic composition of the product methane. This result is expected, given that the strains used in our experiments lack a functional uptake hydrogenase (64) and that nitrogenase itself is not thought to catalyze isotope exchange between water and H_2_ (65). Isotopic exchange is distinct from the hydrogenation of ^2^H_2_, forming ^1^H^2^H, which nitrogenase does catalyze in the presence of N_2_. We note that abiotic hydrogen isotopic equilibration between H_2_-H_2_O, CH_4_-H_2_, and CH_4_-H_2_O is likely too slow to be important at the timescales (∼weeks) and temperatures (≤30°C) of relevance to our experiments (37, 66–68). Our finding that exogenous H_2_ does not alter the isotopic composition of product methane in the R. palustris V- and Fe-only nitrogenase strains is consistent with other reports that the source of protons for carbon monoxide reduction by nitrogenase is water, not hydrogen gas (16). We did not test whether the presence of exogenous H_2_ influences the measured hydrogen isotope fractionation when an uptake hydrogenase is present (69), a mechanism that may be significant for the δ^2^H_H2_ effect on the isotopic composition of methane generated by hydrogenotrophic methanogenesis (48, 56, 57, 59).

**FIG 6:**
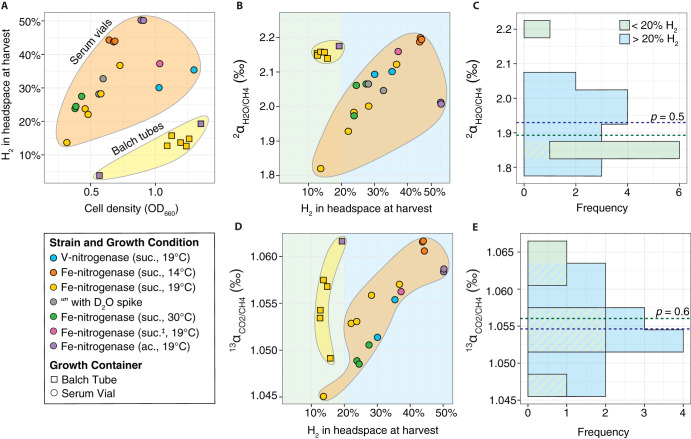
Hydrogen concentration does not alter the isotopic composition of nitrogenase-derived methane. (A) Strains produce hydrogen (H_2_) proportional to growth. Correspondingly, the cultures grown in Balch tubes, which had higher headspace-to-volume ratios, accumulated lower concentrations of hydrogen. Comparison of hydrogen (B) and carbon (D) isotope fractionations between cultures grown in Balch tubes and serum vials shows that hydrogen concentration is not responsible for the variability in fractionation between samples. The methane from cultures harvested at high cell densities in serum vials had a range in isotope fractionation similar to that of methane from cultures grown in Balch tubes despite >2-fold differences in headspace hydrogen concentrations. This is also apparent in histograms C and E, which show that the distribution of isotope fractionation is the same (*P > *0.5) for cultures whose headspace hydrogen concentration at harvest was between 10 and 20% (green) or 20 and 50% H_2_ (blue).

The second line of evidence demonstrates that H_2_ concentration does not influence nitrogenase methane isotope fractionation by comparing the fractionations observed in different growth containers and for the different strains. For a given growth container and strain, cell density and hydrogen concentration are correlated (Fig. 6A; also see the discussion in the supplemental material). However, their respective effects on fractionation can be disentangled by comparing data from Balch tubes (10 ml medium, 17 ml headspace) and serum vials (180 ml medium, 60 ml headspace). As seen in Fig. 6, hydrogen and carbon isotope fractionations in cultures with 10% to 20% H_2_ in the headspace at harvest overlap those of cultures with 20% to 50% H_2_ in the headspace at harvest (*P > *0.5) (Fig. 6C and E). We conclude that fractionation during methane production by nitrogenase is not sensitive to hydrogen concentration over the large range (10% to 50%) tested here. This is compatible with findings that carbon dioxide reduction by Mo-nitrogenase is not competitively inhibited by H_2_ and does not proceed through the same reversible *re* (reductive elimination of H_2_) step as N_2_ reduction (70). The lack of hydrogen partial pressure dependency on fractionation contrasts with some modes of *mcr*-based methanogenesis (2, 43, 48, 54–63).

### Mechanistic implications for nitrogenase.

Determining whether isotope effects are due to equilibrium or kinetic fractionation and under what conditions they are fully expressed can help elucidate the mechanism, intermediates, and reversibility of a reaction. At 20°C, the equilibrium hydrogen isotope fractionation predicted between methane and water, ^2^α_H2O/CH4_, is ∼1.019 (71). This is much smaller than the fractionation observed for nitrogenase (∼2.1), suggesting that kinetic, rather than equilibrium, isotope effects are responsible for the large hydrogen isotope fractionation observed here. This conclusion is consistent with the finding that fractionation of carbon dioxide reduction by nitrogenase is larger at colder temperatures (Fig. 5B and F), which is generally incompatible with an equilibrium isotope effect (72). These results lead us to attribute the fractionation observed here (1.820 ≤ ^2^α_H2O/CH4_ = KIE ≤ 2.199, mean = 2.1) to a kinetic isotope effect (KIE) in which C^1^H_4_ methane production by V- and Fe-only nitrogenase is roughly twice as fast as C^1^H_3_^2^H methane production. We suggest that these new values can help yield insight into the mechanism of CO_2_ reduction by nitrogenase.

**FIG 7:**
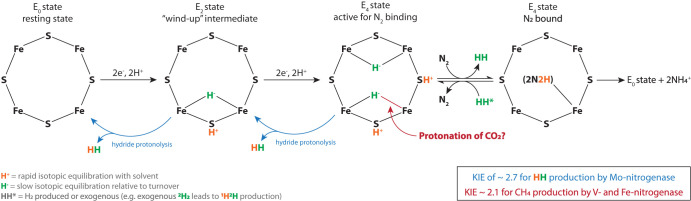
Illustrative schematic highlighting likely proton sources for N_2_ and CO_2_ reduction and H_2_ production by nitrogenase. The different hydrogen kinetic isotope effects (KIEs) for H_2_ production by Mo-nitrogenase compared to CH_4_ production by the V- and Fe-only nitrogenases could be due to different ^1^H selectivities by each isoform or due to different contributions by the protonated thiols (orange) compared to the bridging Fe-hydrides (green) for reduction of different substrates (e.g., N_2_ versus CO_2_). For instance, it has been suggested that CO_2_ can migrate into the Fe-hydride bond (70, 94), although it is not known at which *E_n_* state this might occur or whether this is necessarily the only pathway for CO_2_ reduction to methane. Modified from reference 73 with permission.

There is limited experimental data regarding hydrogen stable isotope fractionation by nitrogenase. The only existing measurements of hydrogen stable isotope fractionation are for H_2_ production (^2^α_H2O/H2_) in the absence of N_2_ by the Mo-nitrogenase (73). Khadka and colleagues (73) used this data as a tool to determine the mechanism of H_2_ loss during activation of the cofactor, a catalytically inefficient reaction that competes with N_2_ reduction (Fig. 7). They demonstrated, experimentally and computationally, that the KIE of ∼2.7 for H_2_ production by Mo-nitrogenase is due to the preference for ^1^H during protonation of the bridging Fe-hydrides by highly acidic, protonated cofactor sulfides.

The mechanism of CO_2_ reduction by nitrogenase is a subject of much study because of its potential industrial application as a renewable fuel source (70, 74, 75, and references therein). Our observation that the hydrogen KIE during methane production (^2^α_H2O/CH4_) for V- and Fe-only nitrogenases is ∼2.1 represents a new experimental constraint for these studies. There are several possible explanations for why the ^2^α_H2O/CH4_ value for alternative nitrogenases is lower than the ^2^α_H2O/H2_ of Mo-nitrogenase (73). For example, it could be that the V- and Fe-only nitrogenases are less selective for ^1^H than the Mo-nitrogenase. The nitrogenase isoforms have different cofactor and amino acid environments, which can alter protonation and substrate selectivity (76–78 and references therein). Alternatively, it could be due to different hydrogen isotope effects for different substrates (e.g., N_2_ versus CO_2_), possibly due to differing contributions by the protonated sulfides, which can exchange with solvent at the timescale relevant to the reaction, compared to the bridging Fe-hydrides, which do not (Fig. 7) (73). It is also possible that proton tunneling, which is generally thought to have a very large KIE (but also see references 96 and 97) and has been proposed to occur in nitrogenase (79), could be contributing to the KIE observed here, although we note that the temperature effect observed here is opposite the predicted effect for tunneling (80, 81). Computational models, which can distinguish the rates of hydrogenation based on ^1^H and ^2^H, and might be able to shed light on the mechanism responsible for the observed fractionation and whether the currently proposed, multistep mechanisms of hydrogenation by nitrogenase (82–85) are compatible with the measured KIE of 2.1. The clumped isotopic composition of methane produced by nitrogenase could also provide additional constraints.

### Environmental relevance.

The carbon and hydrogen isotopes of methane are critical constraints for the attribution of emissions of this potent greenhouse gas to its sources (86). Our characterization of nitrogenase’s biosignature helps refine the space of possible source δ^13^C and δ^2^H values. The characteristic δ^2^H signature of alternative nitrogenases distinguishes them from other microbial and thermogenic methane sources (Fig. 2). At −550‰, the δ^2^H of nitrogenase-derived methane falls well below the lowest values, around −400‰, that have been observed for other biotic and abiotic processes (2, 39).

Given the ubiquity of carbon dioxide in cells and in the environment, it is likely that some methane production is occurring whenever V- and Fe-only nitrogenases are active. However, this flux is orders of magnitude smaller than N_2_ reduction (∼5 × 10^−4^ CH_4_:1 N_2_ for Fe-only nitrogenase in our experiments; data not shown) and can be limited by intracellular energy (10, 33) and presumably DIC availability. The *K_m_* values of the V- and Fe-only nitrogenases for CO_2_ reduction into methane have not been measured, but, based on similar systems, are likely at the high end of environmental DIC concentrations, around 10 or 20 mM (see the discussion in the supplemental material). It will be valuable for future studies to elucidate how variable CH_4_ production stoichiometry by V- and Fe-only nitrogenases is and what factors control this variability.

Even without such data, it is clear that methane production by the V- and Fe-only nitrogenases does not contribute quantitatively to methane production at the global scale (10). For instance, assuming generously that ∼20% of the ∼145 Tg annual terrestrial biological nitrogen fixation flux (∼120 Tg year^−1^ from reference 87, corrected for underestimation by the acetylene reduction assay as described in reference 27) is fixed by Fe-only nitrogenase, and recognizing that methane itself is a minor by-product of dinitrogen reduction (using 5 × 10^−4^ CH_4_:1 N_2_), the resultant ∼0.01 Tg year^−1^ is negligible compared to total methane emissions of ∼560 Tg year^−1^ (88).

**FIG 8:**
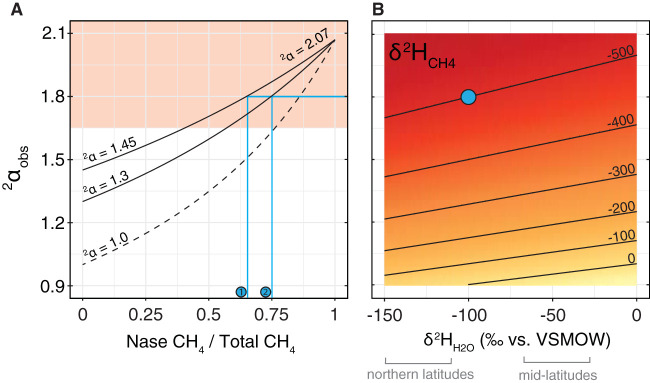
Observed fractionation (A) and methane isotopic composition (B) for multiple methane sources. Panel A shows the apparent fractionation (^2^α_H2O/CH4_) between water and methane in an environment with co-occurring production from nitrogenase (^2^α_H2O/CH4_ = 2.07; *x* axis shows the relative contribution of nitrogenase to the total methane pool) and a second source with ^2^α_H2O/CH4_ = 1.0 (dashed line; no expressed fractionation), ^2^α_H2O/CH4_ = 1.3 (broadly representative of hydrogenotrophic methanogenesis [34]), or ^2^α_H2O/CH4_ = 1.45 (broadly representative of acetoclastic methanogenesis [34]). Panel B shows the calculated methane isotopic composition δ^2^H_CH4_ given the isotopic composition of local source water (*x* axis) and the measured fractionation between water and methane (^2^α_H2O/CH4_; *y* axis). For example, for a sample with ^2^α_obs_ = 1.8 (e.g., when δ^2^H_H2O_ = −100‰, δ^2^H_CH4_ = −500‰, shown as a blue circle in panel B), the model predicts that ≥70% of the methane is produced by nitrogenase, depending on whether the competing source of methane is fermentative (blue line 1, ∼70%) or hydrogenotrophic (blue line 2, ∼75%).

Nonetheless, we hypothesize that the alternative nitrogenases’ large hydrogen isotope fractionations could influence methane isotopic composition, and act as a biomarker for alternative nitrogenase activity, in nitrogen-limited environments with low methanogenesis rates and high alternative nitrogenase activity. We developed a simple isotope-mixing model to quantitatively determine the extent to which stable isotopes can attribute methane production to alternative nitrogenase activity in environments with multiple sources (Fig. 8). The model calculates the net ^2^α_H2O/CH4_ and δ^2^H of the mixed methane pool given the local water isotopic composition, the fraction of total methane generated by nitrogenase, and net ^2^α_H2O/CH4_ fractionation values for methane generated by other physiological pathways, not accounting for possible contributions from δ^2^H_H2_ or δ^2^H_acetate_. Based on a compilation of ^2^α_H2O/CH4_ values measured in diverse pure-culture experiments (48), we suggest that ^2^α_H2O/CH4_ of ≥1.65 (shown in red in Fig. 8A) would provide evidence for alternative nitrogenase activity in natural samples. Using this constraint, the isotopic mixing model demonstrates that methane stable isotopes can identify alternative nitrogenase activity as long as the rate of methane production from nitrogenase is faster than or comparable to that of other methane-producing pathways but not when it is slower.

The controls on alternative nitrogenase activity are not fully understood (e.g., references 25, 27, 89, and 98), although new tools (27, 34) are rapidly advancing our understanding of their distribution. It is now well established that alternative nitrogenases are favored under conditions of low Mo availability (28, 90), although their activity has been observed in some sedimentary environments that appeared to be Mo-replete as well (26, 27). Aerobic soils, cyanolichens, mosses and other biocrusts, lake and marine waters (8, 91), or sediment systems with high sulfate concentrations, where sulfate reducers generally outcompete methanogens for substrates (3, 92), are possible targets to test where and when alternative nitrogenases are active using methane stable isotopes (10). Our results present an exciting avenue for future research aimed at constraining the importance of nitrogenase to methane production in environments with low activity of canonical methanogens and at illuminating the mechanism(s) of nitrogenase CO_2_ reduction.

### Conclusions.

Nitrogenases are important enzymes in the global nitrogen cycle. The curious observation that two of the three isoforms, V- and Fe-only, produce small quantities of methane from carbon dioxide led us investigate the isotopic composition of nitrogenase-derived methane as it compares to other biogenic and abiogenic sources in nature. Here, we show that the δ^2^H of nitrogenase-derived methane can be as low as −550‰. This is significantly lower than the δ^2^H of methane from all other known processes. This result provides new experimental constraints on the mechanism of the nitrogenase enzyme and demonstrates that significantly depleted hydrogen stable isotopic composition constitutes a passive biosignature of V- and Fe-only nitrogenase-derived methane. This isotopic fingerprint offers a means to probe the contribution of alternative nitrogen fixation and nitrogenase methane emissions on Earth and beyond.

## MATERIALS AND METHODS

### Bacterial cultures.

Rhodopseudomonas palustris strains CGA766 (V-nitrogenase strain; genotype, Δ*nifH nifD*::*Tn5* Δ*anfA*) and CGA755 (Fe-only nitrogenase strain; genotype, Δ*nifH* Δ*vnfH*) were grown in batch cultures at 14, 19, and 30°C and ∼90 μmol photons m^−2^ s^−1^ under anaerobic photoheterotrophic conditions in defined nitrogen-fixing medium with 2.5 μM Fe, 100 nM Mo, 10 μM V, Wolfe’s vitamin solution, 0.0005% yeast extract, and either 10 mM succinate or 20 mM acetate (34, 41, 42). Where applicable, the δ^2^H of the growth medium was manipulated by adding 99.9% purity D_2_O (Cambridge Isotope Laboratories, Inc.) to the growth medium. Bacterial growth was monitored by optical density at 660 nm (OD_660_) using a Genesys 20 visible spectrophotometer (Thermo Fisher Scientific) and converted to cell density using the empirically observed relationship cells ml^−1^ = 2.29 × 10^9^ × OD_660_.

### Analytical.

Methane concentrations in the culture headspaces were measured either on a Peak Performer 1 gas chromatograph with N_2_ carrier gas (Peak Laboratories) or on a GC-8A with He carrier gas (column, Supelco HayeSep N; column temperature, 80°C; detector temperature, 150°C; Shimadzu Instruments) with flame ionization detectors. Calibration curves were made by sequentially diluting 100 ppm or 1% CH_4_ standards with N_2_ in a 10-ml syringe with a Luer lock and, like the samples, loading 1 ml onto the instrument using an injection loop. Hydrogen and carbon dioxide gas concentrations were measured using gas chromatography with a thermal conductivity detector (GC-8AIT TCD; column, Restek ShinCarbon ST; column temperature, 100°C; detector temperature, 150°C; Shimadzu Instruments) with N_2_ as the carrier gas. Dissolved methane was not quantified. We note that not all variables were measured in all samples, and that the raw data points used for all the figures and calculations in this report are available in the supplemental material.

### Stable isotope measurements.

Methane samples were analyzed for δ^2^H and δ^13^C at the UC Davis Stable Isotope Facility. Depending on the methane concentration, samples were collected either in preevacuated 12-ml soda glass vials (839W; Labco Limited) or diluted in He-flushed vials. Because sample methane δ^2^H was depleted relative to the lowest standard available at the UC Davis Stable Isotope Facility (−276‰), a dilution series of a single sample was measured, and the resulting linearity correction was applied to all samples (calculations included in Supplemental File 2). The constant hydrogen isotope fractionation observed for Fe-only nitrogenase over a >500‰ range in δ^2^H suggests that the analytical methods employed are robust (Fig. 3). Samples for δ^13^C analysis of CO_2_ were collected in the same manner as those for methane. Samples for δ^13^C of DIC were collected in He-flushed vials that contained 1 ml of concentrated high-performance liquid chromatography (HPLC)-grade phosphoric acid (85%; Fisher Chemical). At the UC Davis Stable Isotope Facility, the δ^2^H_CH4_, δ^13^C_CH4_, δ^13^C_CO2_, and δ^13^C_DIC_ samples were measured on a Delta V Plus IRMS (Thermo Scientific, Bremen, Germany) coupled to a Gas Bench II system. Water δ^2^H samples were collected by filtering growth medium (0.22 μm) at the end of the experiment and storing at −20°C. For analysis, samples were thawed, and 1.4 to 1.5 ml was aliquoted into 2-ml soda glass vials (National C4010-1W with C4010-40A caps; Thermo Scientific) and shipped on ice or at room temperature overnight to the UC Davis Stable Isotope Facility, where they were measured on a Laser Water Isotope Analyzer V2 (Los Gatos Research, Inc.). Biomass and substrate δ^13^C were measured in the Zhang stable isotope laboratory at Princeton as described previously (42) on a Vario ISOTOPE select (Elementar Isoprime). The standard deviation of standard material replicates were <1‰ for δ^2^H_H2O_, <2‰ for δ^2^H_CH4_, <0.2‰ for δ^13^C_CH4_ (>10 ppm), <0.2‰ for δ^13^C_CO2_ and δ^13^C_DIC_, and <0.1‰ for δ^13^C_biomass_.

### Isotope nomenclature.

Hydrogen and carbon isotopes are expressed using delta notation relative to Vienna Standard Mean Ocean Water (VSMOW) and Vienna Pee Dee Belemnite (VPDB), respectively:

R2=H2/H1

R13=C13/C12

δ2H=1,000×[(H2/Hsample1)/(H2/HVSMOW1)−1]=1,000×(Rsample2/RVSMOW2−1)

δ2C=1,000×[(C/13Csample12)/(C/13CVPDB12)−1]=1,000×(Rsample13/RVPDB13−1)

Apparent CO_2_-CH_4_ and water-CH_4_ isotope fractionation factors were calculated as substrate over product using the equations

αCO2/CH413=RCO213/RCH413=(δ13CCO2+1,000)/(δ13CCH4+1,000)

αH2O/CH42=RH2O2/RCH42=(δ2HH2O+1,000)/(δ2HCH4+1,000)

ε = (α−1)×1,000‰

In this work, errors represent the standard errors of multiple biological replicates.

### Isotope mixing model.

To determine under what conditions the methane isotopic composition can be used as a biosignature for alternative nitrogenase activity, we developed a mixing model that calculates the fractionation and isotopic composition of methane produced by multiple sources (Fig. 8). We used the following parameters: ^2^α_Nase_ = 2.07; δ^2^H_H2O_ = −40‰ versus VSMOW as representative of the mid-latitudes and −150‰ versus SVMOW as representative of northern latitudes; and *k* = the fraction of total methane produced by nitrogenase. For fermentative methanogenesis, the model assumes that all protons ultimately derive from local water. The observed fractionation and isotopic composition were calculated using the equations

FNase/mcrCH42=RNase/mcrCH42/(1+R2Nase/mcrCH4)

FCH42=k×FNaseCH42+(1−k)×FmcrCH42

### Data availability.

Individual data points are available in Supplemental File 2. In addition to Supplemental File 2, these data are preserved in FigShare (https://figshare.com/articles/Data_associated_with_large_hydrogen_isotope_fractionations_distinguish_nitrogenase-derived_methane_from_other_sources_/12343997).

## Supplementary Material

Supplemental file 1

Supplemental file 2
