# Supplementary material for: Large Hydrogen Isotope Fractionation Distinguishes Nitrogenase-Derived Methane from Other Methane Sources
Source: Appl Environ Microbiol. 2020 Sep 17;86(19):e00849-20. doi: 10.1128/AEM.00849-20 (PMC7499036; doi:10.1128/AEM.00849-20)
Supplement: Supplemental file 1 [file AEM.00849-20-s0001.pdf]

Supporting Information for:

Large hydrogen isotope fractionations distinguish nitrogenase-derived methane from other sources

Katja E. Luxem,[1,2*] William D. Leavitt,[3,4,5] Xinning Zhang[1,2*]

[1]Dept. of Geosciences, [2]Princeton Environmental Institute, Princeton University; [3]Dept. of Earth Sciences, [4]Dept. of Chemistry, [5]Dept. of Biological Sciences, Dartmouth College

Relationship between dissolved and headspace H_2 concentrations

The conclusion that fractionation during methane production by nitrogenase does not depend on the concentration of H_2 is based on measurements of the percentage of H_2 in the headspace at harvest (Fig. 6). However, the *R. palustris* cultures generally produce some overpressure during growth (S.I. Table; Tab: "Compiled Data"). According to Henry's Law, the dissolved H_2 concentration, which is most representative of the conditions experienced by the cells, depends on the product of the partial pressure (i.e. percentage of H_2) and the total pressure (i.e. overpressure). Thus, our conclusion would be undermined if the overpressure in the balch tubes were > twice as large as the overpressure in the serum vials (i.e. ≥ ~ 4 atm). We did not record the overpressure in the sampled balch tubes, but our measurements of other cultures grown in balch tubes suggest that it is unlikely that overpressures would have been > 3 atm. Moreover, from a theoretical perspective, the balch tubes had a lower liquid : headspace ratio than the serum vials, therefore the expectation would be that, if there were a difference in overpressure between the balch tubes and serum vials, it would be that the balch tubes would be less overpressurized than the serum vials at the same cell culture density (OD_{660}).

Possible effect of lower DIC concentration on fractionation: extrapolation and assumptions

With the existing dataset, it is not possible to determine whether DIC concentration, growth phase or cell density is responsible for the correlation with carbon and hydrogen stable isotope fractionation seen in Figs. 5 C,D,G, and H. However, if DIC concentration were the causative factor, it would suggest that, in environments with low DIC concentrations or in autotrophs with intracellular DIC depletion, the hydrogen stable isotope composition of methane produced by nitrogenase might be less depleted than reported here. For purposes of discussion below, we assume that DIC, rather than growth phase or cell density, is the causative factor controlling the ~0.01 shift in carbon (Fig. 5G, H) and ~0.25 shift in hydrogen (Figs. 5C, D) stable isotope fractionation during growth.

The anaerobic medium for *R. palustris* is prepared by degassing and purging with N_2 gas, resulting in very low DIC concentrations before inoculation. DIC builds up during logarithmic growth, plateaus, and is progressively consumed as stationary phase progresses. In the *R. palustris* cultures, DIC concentrations ranged from approximately 2.4 to 12 mM at harvest, *i.e.* when methane was sampled. These concentrations are at the high end of those found in natural environments, typically from 0.1 to 5 mM in rivers and lakes and from 0.5 to 8 mM in groundwater (1). Due to DIC production during photoheterotrophic growth by *R. palustris* and the detection limits for measurements of methane isotopic composition, it was not possible to measure fractionation at lower DIC concentrations. To predict whether fractionation would be significantly different if DIC concentrations had been an order of magnitude lower, the trend observed in Figs. 5D and H was extrapolated (Fig. S1AB). Linear extrapolations (R^2 = 0.98) suggest that hydrogen isotope fractionation is around ${}^{2}\alpha_{H2O/CH4} \sim 1.7$ and carbon isotope fractionation around ${}^{13}\alpha_{CO2/CH4} \sim 1.038$ at near-zero DIC concentrations. Though lower, these fractionations are not substantially

outside of the range reported here ($1.820 \leq {}^{2}\alpha_{H2O/CH4} \leq 2.199$; $1.030 \leq {}^{13}\alpha_{CO2/CH4} \leq 1.080$) and would not materially alter our mechanistic interpretation or ability to detect this alternative nitrogenase biosignature in the environment.

However, we cannot exclude the possibility that the linear extrapolation overestimates fractionation at low DIC concentrations. In general, substrate concentration effects on fractionation are due to a change in the thermodynamic driving force and reaction reversibility. It is not yet known whether CO_2, HCO_3^- or CO_3^{2-} is the substrate for nitrogenase (2) and the K_m values for the reduction of CO_2 to CH_4 by the V- and Fe-only nitrogenases have not been measured. An early study with purified Mo-nitrogenase found a K_m of ~40 mM for [CO_2 + HCO_3^-] reduction to CO, corresponding to 23 mM if CO_2 is assumed to be the active species (3). Experiments with a mutant Mo-nitrogenase yielded a K_m with a similar order of magnitude, of 16 mM for $NaHCO_3$ or 7.9 mM for CO_2 depending on which is assumed to be the active substrate (4). Assuming that the K_m values for CO_2 reduction into CH_4 by the V- and Fe-only nitrogenases are of a similar order of magnitude, this would imply that the concentrations in our experiments (~2.5 to 12 mM) are sub-saturated. In the absence of other constraints, being in the range of substrate concentrations with linear rate dependence is encouraging and lends some support to the validity of the linear extrapolation between substrate concentration and fractionation. However, future pure culture experiments with autotrophic growth conditions or chemostat conditions will be necessary to test this with certainty.

Fig. S1. Linear best fit lines for extrapolation of fractionation to lower DIC concentrations. This correlation assumes that DIC concentration, rather than growth phase or cell density, is responsible for the correlation.

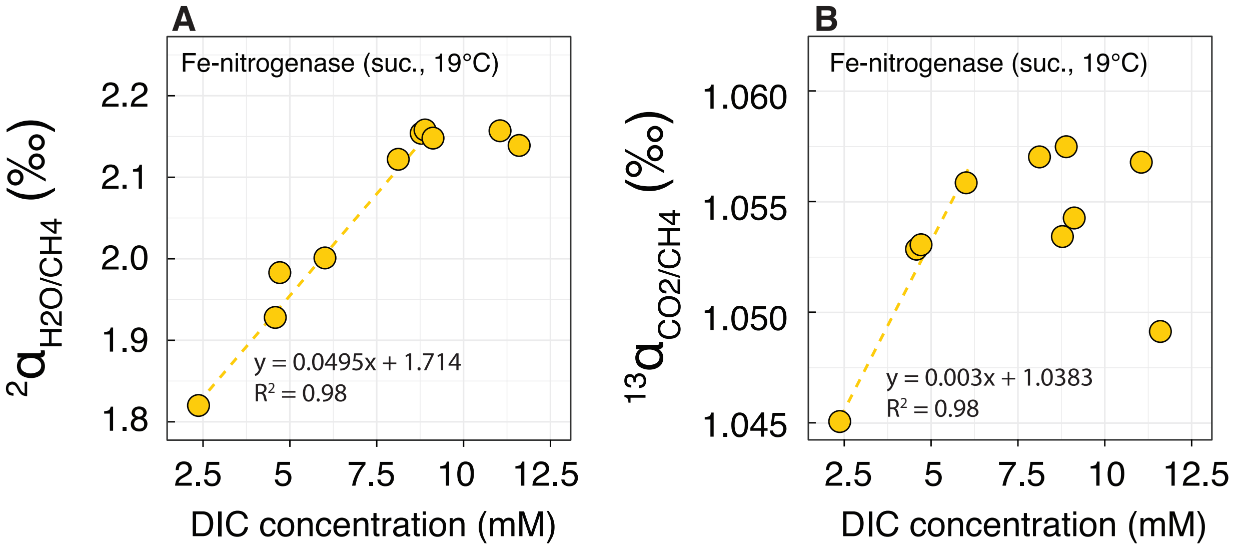

Supporting Information References

1. Stumm W, Morgan JJ. 1995. Aquatic Chemistry : Chemical Equilibria and Rates in Natural Waters. John Wiley & Sons, Incorporated, Somerset, United States.
2. Seefeldt LC, Yang Z-Y, Lukoyanov DA, Harris DF, Dean DR, Raugei S, Hoffman BM. 2020. Reduction of Substrates by Nitrogenases. Chem Rev.
3. Seefeldt LC, Rasche ME, Ensign SA. 1995. Carbonyl sulfide and carbon dioxide as new substrates, and carbon disulfide as a new inhibitor, of nitrogenase. Biochemistry 34:5382–5389.
4. Yang Z-Y, Moure VR, Dean DR, Seefeldt LC. 2012. Carbon dioxide reduction to methane and coupling with acetylene to form propylene catalyzed by remodeled nitrogenase. Proc Natl Acad Sci 109:19644.
